# Special Hospitals: Robbers of the Poor

**Published:** 1895-02-23

**Authors:** 


### The Institutional Workshop.

### HOSPITAL FINANCE.

### SPECIAL HOSPITALS; ROBBERS OF THE POOR.

The cry from South London for more hospital accommodation, the similar lament from the great and growing population of the north-eastern districts, the want of hospital accommodation in the western outgrowths of London—a want which has been held to justify the patronage by the Hospital Sunday Fund of trumpery institutions which but for such want would certainly never have received a grant—all point to the fact that while London is growing the general hospital accommodation is not growing in proportion to its wants, and that to provide the deserving poor with the benefits of modern advances in medicine and surgery, benefits which they cannot obtain except in hospitals, more hospitals are required.

Yet, coincident with all this we find ourselves face to face with the uncomfortable fact that several of our large general hospitals are partly closed, that their wards stand empty, that their healing power is not working to the full, that their accessory buildings, such as kitchens, washhouses, dispensaries, mortuaries, &c., planned for complete establishments, are only partly used, and that their fixed expenses in the way of secretaries, matrons, porters, and all those other matters which are practically the same for both large and small institutions, are just the same as if they were fully occupied.

The wastefulness of the arrangement is obvious, and in each case we are told that the cause is want of money. According to Mr. Burdett's statistics, however, published in the *Times*, the fount of charity still runs, and money is being given to hospitals and charities at large in an undiminished stream. Where then does it go? Why in the midst of such abundance do we find St. Thomas's proclaiming its empty coffers and its unopened wards; why should both it and Guy's, while declining the principle of payment from all who can pay, and maintaining the pose of charity, yet use up the beds, which by their own hypothesis belong to the poor, so as to earn an honest penny? Why should the Metropolitan Hospital stand with its wards empty, naked, and unashamed, and the Temperance Hospital, the exponent of a sect so powerful that it never ceases to proclaim its influence in politics, remain with its wards unfilled and its missionary usefulness only partially employed?

The answer is that the stream of charity is being diverted, tapped at the fountain head and turned into less useful channels. While the people are calling out for hospital treatment, and hospital managers are pointing to their empty coffers as an excuse for their empty beds, the special hospitals thrive, and it is hard to avoid the conclusion that so far as they divert the stream of charity they are robbers of the poor.

We are not concerned to deny that some of the special hospitals had some excuse for their inception. One need not to be very old to remember the time when all specialism, except that of the dentist, was looked on as a medical heresy, and when the specialist was dealt with almost like a heretic of old. Denied the opportunity of working on their own line in the general hospitals, and openly branded as self-seekers, it was not to be wondered that energetic men sought new fields and established so-called hospitals for themselves. Often poor, meagre, and ill-found, but always worked with energy and enthusiasm, as the first revolts from fossil conservatism usually are, they forced their way and served their purpose. But so far as the public is concerned the great purpose which they served was not the pouring of wealth into the pockets of their founders, nor the benefit which their patients derived from them, nor even the scientific investigations carried out within their walls, but the breaking down of the old idea that every doctor was a jack of all trades, and the introduction, into every hospital of note, of departments for the treatment and the teaching of the various specialties. Special hospitals have served their purpose, and, so far as the good of the public is concerned, they have done all the good they are capable of. What is now wanted is the grouping of the specialties as departments of general hospitals, and the abolition of that independent self-seeking specialism which was the origin, and still is the vice, of so many special hospitals.

The total income of the special hospitals is somewhat startling, and, when put side by side with that of the general hospitals, shows how large a drain they must be upon the fund on which the hospitals live.

Thus, according to "Burdett's Hospital Annual" for 1894, the income of the general hospitals in London, both connected and unconnected with medical schools, was in 1892 £524,249, while the income of the special hospitals, excluding fever hospitals, was £242,617. That is, the special hospitals in London absorbed an income nearly half as large as that of all the general hospitals put together.

But if we take out from the list of special hospitals those which are doing work which is not usually accepted by the general hospitals, such as all the consumption hospitals, all the lying-in hospitals, and the Lock Hospital, as well as those for fever, we still have a total income of £159,419 going to institutions which are doing the same work as that which is being done in the general hospitals.

This, however, does not express the whole magnitude of the evil. There are various semi-private institutions for the treatment of various diseases, which, although unworthy of the name of hospitals, or of being included in Mr. Burdett's lists, still absorb subscriptions.

Let us then consider what all this means. There are in London alone at least 24 institutions, obtaining in one way or another at least £159,419 per annum, which they expend in doing the same class of work as is done by the general hospitals. For the doing of this we may presume that there are at least 24 secretaries, 24 matrons, 24 head cooks, besides 24 offices tc keep up, 24 boards to meet, 24 advertisements on every occasion, and other expenses in proportion.

The waste is frightful; for it is not as if the existence of these special hospitals in any way took off from the general hospitals the necessity of providing special departments. Every school hospital has its special arrangements for the treatment of such diseases of eyes, ears, throats, skin, women, children, &c., as they can find, while the cream of the cases which would be so useful for clinical instruction drift off to the special hospitals, attracted by the name of the disease from which they think they suffer. The whole thing is a muddle, there seems no redeeming point about it; the cases so useful for clinical instruction pass out of the ken of the student to hospitals to which he can only gain access by further fees, while the money which would provide for them in the general hospitals drifts away to special institutions, to be used in many cases, it is to be feared, for the relief of those who can hardly be called poor.

